# Determination of Flavonoids and Carotenoids and Their Contributions to Various Colors of Rose Cultivars (*Rosa* spp.)

**DOI:** 10.3389/fpls.2019.00123

**Published:** 2019-02-12

**Authors:** Huihua Wan, Chao Yu, Yu Han, Xuelian Guo, Le Luo, Huitang Pan, Tangchun Zheng, Jia Wang, Tangren Cheng, Qixiang Zhang

**Affiliations:** ^1^Beijing Key Laboratory of Ornamental Plants Germplasm Innovation and Molecular Breeding, National Engineering Research Center for Floriculture, Beijing Laboratory of Urban and Rural Ecological Environment, Engineering Research Center of Landscape Environment of Ministry of Education, Key Laboratory of Genetics and Breeding in Forest Trees and Ornamental Plants of Ministry of Education, School of Landscape Architecture, Beijing Forestry University, Beijing, China; ^2^Beijing Advanced Innovation Center for Tree Breeding by Molecular Design, Beijing Forestry University, Beijing, China

**Keywords:** rose, petals, color, anthocyanins, flavonols, carotenoids

## Abstract

Rose is one of the most valuable ornamental crops worldwide. In this study, the composition of hydrophilic and lipophilic pigments in petals of six rose cultivars at seven developing stages was investigated using high performance liquid chromatography and mass spectrometry. Four anthocyanins, 20 flavonols, and 10 carotenoids were detected in petals of tested cultivars. Major individual anthocyanin, flavonol, and carotenoid were cyanidin/pelargonidin 3,5-diglucoside, kaempferol 3-*O*-rhamnoside, and (9*Z*)-violaxanthin, respectively. Significant differences were observed in pigments content in petals of different rose cultivars. The yellow petals of YI and GC exhibited no to very small amounts of anthocyanins, moderate amount of total flavonols, and highest content of total carotenoids. Similarly, pink petals of PF, WQ, and YX showed average concentration of total anthocyanins, highest concentration of total flavonols, and small amount of carotenoids. Further, orange petals of CH showed highest content of total anthocyanins, lowest content of total flavonols, and average content of total carotenoids. Correlation analysis demonstrated that there were many pigments influencing petal colors. Moreover, multiple linear regression indicated that pelargonidin 3,5-diglucoside, total anthocyanins and (9*Z*)-violaxanthin were the major factors. In addition, this study showed that orange cultivar CH, pink cultivar PF and yellow cultivar YI can have great potential as a natural source for the extraction of pelargonidin 3-*O*-glucoside, kaempferol 3-*O*-rhamnoside, and (9*Z*)-violaxanthin, respectively. These investigations would contribute toward understanding the mechanism on the development of flower colors and provide a theoretical basis for the breeding of rose with specific color.

## Introduction

*Rosa* spp., a member of Rosaceae family, is one of the most expensive ornamental crops worldwide, which is used as cut flowers, garden ornamentals, and potted plants (Hibrand Saint-Oyant et al., 2018; Raymond et al., 2018). Flower color is the predominant characteristic of modern rose cultivars due to its contribution toward the pleasant appearance of roses. The mixture and blending of anthocyanins, flavonols, and carotenoids provide multiple colors to rose petals (Eugster and Märki-Fischer, 1991; Bendahmane et al., 2013). Due to various biological activities of these compounds, rose petals have been widely used in food, drug, and cosmetics (Guimarães et al., 2010; Lee et al., 2011; Prata et al., 2017).

Flavonoids are a large group of phenylpropanoids, including anthocyanins, flavonols and flavone, and other compounds. Various phytochemical studies have focused on the composition of anthocyanins, which impart pink to red hues to rose flowers (Biolley and Jay, 1993; Mikanagi et al., 1995, 2000; Lee et al., 2011). Three major anthocyanidins, such as pelargonidin, cyanidin, and peonidin have been found in roses. These compounds are stabilized by glucosylation mostly as anthocyanidin3,5-*O*-diglucosides, and slightly as anthocyanidin 3-*O*-glucosides (Biolley and Jay, 1993; Mikanagi et al., 2000; Ogata et al., 2005). Flavonols are pale yellow or colorless compounds, which are not only the co-pigments of anthocyanins, but also could absorb ultraviolet light to protect petals and attract pollinating insects (Tanaka et al., 2008). Several reports have reported that the predominant flavonol aglycones were kaempferol and quercetin in rose petals (Mikanagi et al., 1995; Raymond et al., 1995; Kumar et al., 2009; Sarangowa et al., 2014).

Carotenoids, a class of isoprenoid compounds, are natural pigments that provide yellow to orange and red hues to plants. The compositions of carotenoids in flowers vary widely among plant species and cultivars. Pale to deep yellow flowers of most plants mainly accumulate xanthophylls (Zhu et al., 2010; Ohmiya, 2011). However, petals of some plants, such as *Adonis aestivalis* and *A. annua* can synthesize high amount of astaxanthin, resulting in their blood-red color appearance (Cunningham and Gantt, 2005). Thus, carotenoids enable plants to exhibit various flower colors. However, in rose petals, various studies focused on carotenoids composition in yellow or related rose varieties and species (Eugster and Märki-Fischer, 1991; Wan et al., 2018).

Anthocyanins, flavonols, and carotenoids are important pigments that have always existed in same plant parts and make them colorful. Anthocyanins are water-soluble, and stored in vacuoles (Tanaka et al., 2008). However, carotenoids are fat-soluble, and stored in chromoplasts (Li and Yuan, 2013). Even though co-pigmentation between anthocyanins and flavonols has been extensively investigated in roses (Biolley and Jay, 1993), interactions between anthocyanins and carotenoids remain unclear in the process of coloration of rose petals. So far, there are few reports that investigated the contributions of anthocyanins, flavonols, and carotenoids to different rose flower colors (Eugster and Märki-Fischer, 1991).

The aim of this study was to explore the possible relationship between anthocyanins, flavonols, carotenoids, and rose flower color. In this context, we identified and quantified anthocyanins, flavonols, and carotenoids in the petals of six different colored rose cultivars at various developing stages by HPLC and MS. In addition, we anticipate that this study will help specific cultivars to be identified as potential valuable resources for the extraction of anthocyanins, flavonols, or carotenoids.

## Materials and Methods

### Reagents and Chemicals

All reagents and chemicals used in this study were of HPLC grade except for sodium chloride (NaCl) and potassium hydroxide (KOH), which were analytical grade and procured from Beijing Chemical Reagent, Co. (Beijing, China). Methanol, formic acid, methyl tert-butyl ether (MTBE), hexane and ether were purchased from Fisher (Fair, United States). Trifluoroacetic acid (TFA), butylhydroxytoluene (BHT) and standards, such as cyanidin 3,5-*O*-diglucoside, pelargonidin 3,5-*O*-diglucoside, quercetin 3-*O*-glucoside, kaempferol 3-*O*-rutinoside, kaempferol 3-*O*-glucoside, kaempferol, and β-carotene were procured from Sigma-Aldrich (St. Louis, MO, United States). Remaining standards, including violaxanthin, antheraxanthin, and zeaxanthin were purchased from CaroteNature (Ostermundigen, Switzerland). HPLC grade deionized water was produced with a Pall Cascada LS Ultra Pure Water system (18.2 MΩ cm, East Hills, NY, United States).

### Plant Materials and Sample Collection

In this study, six *Rosa* cultivars were selected based on their flower color that included two yellow flowering cultivars, ‘Yellow Island’ (YI) and ‘Garden City’ (GC), three pink flowering cultivars, ‘Yunzheng Xiawei’ (YX), ‘Wangri qinghuai’ (WQ) and ‘Pink Fan’ (PF), and one orange cultivar ‘Chacok’ (CH). All plants were grown in experimental greenhouses at a breeding nursery of China National Engineering Research Center for Floriculture (Beijing, 40°02′ N, 115°50′ E). Rose flowers were harvested in June, 2015 at seven developing stages as described before (Wang et al., 2004). For each flower, the outermost and innermost petals were discarded. Remaining petals were used for the study. Basal part of each petal was discarded, whose color was distinct from the rest of the petal. Then, processed petals were rapidly frozen and stored at -80°C until the pigment extraction and analysis. Other fresh flowers were used to perform color analysis.

### Color Analysis

The colors of rose flowers were measured by a spectrophotometer NF555 (Nippon Denshoku, Japan). For each flower, five petals were randomly selected except the outermost and innermost layer. The selected petals were then measured at the mid-point on the both adaxial and abaxial surface under Illuminant C and viewing angle of 2°. The values of *L*^∗^, *a*^∗^, *b*^∗^ C^∗^, and *h* were collected and processed using ColorMate software (version 5) (Wan et al., 2015).

### Extraction and HPLC Analysis of Anthocyanins and Flavonols

The extraction of anthocyanins and flavonols were performed as previously described (Wan et al., 2018). About 0.2 g powder [fresh weight (FW)] of all samples was used for pigment extraction with 1.8 mL methanol: water: methane acid: trifluoroacetic acid (70:27:2:1, v/v/v/v) using ultrasonication at 20°C for 30 min. HPLC analysis was outperformed on Waters 2695 system as described by Wan et al. (2018). The wavelength was set at 520 nm for anthocyanin and 350 nm for flavonols. Injection volume and flow rate was set at 10 μL and 0.5 mL/min, respectively. Flavonols and anthocyanins were quantitatively analyzed by external standard method. Calibration equations for cyanidin 3,5-diglucoside and pelargonidin 3,5-diglucoside were y = 32774x – 791.43 (*R*^2^ = 0.9999) and y = 23939x – 15109 (*R*^2^ = 0.9999), respectively.

### LC–MS Analysis for the Identification of Anthocyanin

LC–MS analysis was performed on same HPLC system after coupling to LC-MSD trap VL ion-trap mass spectrometer (Agilent, United States) equipped with an ESI. The mass signal dimension was 50–1100 m/z. Anthocyanins were ionized in positive mode. LC–MS analytical conditions were set as follows: scan range: 50–1100 m/z; capillary voltage: 4000 V; nebulization pressure: 241.3 kPa; drying gas (nitrogen); temperature: 350°C; flow rate: 8.0 L/min; capillary exit voltage: 116.7 V; capillary offset voltage: 75.9 V.

### Extraction of Carotenoids and HPLC Analysis

Carotenoids were extracted as described by Wan et al. (2018). Briefly, powder samples of 0.2–0.3 g (FW) was extracted with methanol, and then extracted with hexane. Next, the mixture was washed by 2 mL of 10% NaCl solution (w/v). Subsequently, organic phase was collected and the bottom layer was re-extracted with 2 mL of hexane/ether (3:1, v/v) until powder became colorless. Solvent extracts were concentrated using a pressured gas blowing concentrator. Dried carotenoids were saponified with 6% KOH solution in methanol (w/v). Then, 1 mL of 10% NaCl (w/v) was added, and mixture was extracted with hexane/ether (3:1, v/v) until it was colorless. Extracted organic solutions were concentrated to dryness in a vacuum concentrator. The dried carotenoids were dissolved in 1 mL of methanol/MTBE (1:1, v/v) prior to HPLC analysis. To avoid carotenoid degradation, 0.1% BHT (w/v) was added to the solvent extracts. HPLC analysis of carotenoids was performed as described by Wibowo et al. (2015). Flow rate was set at 1.0 mL/min and injection volume was 20 μL. Chromatograms were extracted at 450 nm.

### Statistical Analysis

Data was analyzed using IBM SPSS Statistics (version 23.0, Chicago, IL, United States). All assays were performed in triplicate. Flower diameters, color parameters, and pigment contents in petals of six cultivars at seven developing stages were compared by analysis of variance (ANOVA) combined with Duncan’s multiple range tests. Correlation analyses and multiple linear regression (MLR) analyses were performed to estimate the relationship between color parameters and pigment contents.

## Results

### Flower Development

In the tested cultivars, rose flower increased in size throughout development with significant differences in flower diameter observed at different stages (*P* < 0.05, Table 1). Among six cultivars, GC and PF showed largest flower diameter at S4-7, while WQ and CH exhibited smallest flower diameter at S5-7 (Table 1).

**Table 1:** Flower diameter of six cultivars at different developing Stages (S1–S7).

Stage	Flower diameter (mm)					
	
	YI	GC	YX	WQ	PF	CH
S1	16.98 ± 0.92*e*^a^	15.85 ± 0.67*e*	12.86 ± 0.62*e*	12.8 ± 0.68*e*	15.64 ± 2.61*f*	13.08 ± 0.20*f*
S2	21.76 ± 0.88*d*	21.23 ± 1.10*d*	13.26 ± 0.33*d*	16.08 ± 1.61*d*	17.6 ± 2.68*f*	14.43 ± 0.34*f*
S3	23.34 ± 2.53*d*	21.98 ± 1.44*d*	17.58 ± 2.79*d*	22.51 ± 3.84*d*	20.75 ± 2.33*e*	14.56 ± 0.24*e*
S4	46.99 ± 5.43*c*	71.23 ± 4.64*c*	38.16 ± 1.05*c*	38.66 ± 3.91*c*	79.33 ± 8.14*d*	45.25 ± 8.34*d*
S5	68.3 ± 0.80*b*	100.14 ± 1.82*b*	73.88 ± 2.19*b*	60.56 ± 3.54*b*	91.53 ± 10.86*c*	64.43 ± 3.76*c*
S6	88.86 ± 1.59*a*	104.56 ± 2.97*a*	82.8 ± 1.04*a*	73.83 ± 6.80*a*	97.13 ± 12.36*b*	77.45 ± 1.23*b*
S7	91.85 ± 0.20*a*	117.32 ± 3.23*a*	88.71 ± 3.19*a*	81.31 ± 4.88*a*	110.8 ± 12.96*a*	80.99 ± 2.69*a*


*^*a*^Each value presents the mean ± standard deviation (SD) of three independent replicates. Different letters represent significant (*P* < 0.05) differences between means according to analysis of variance (ANOVA) combined with duncan’s multiple range test.*

**FIGURE 1:**
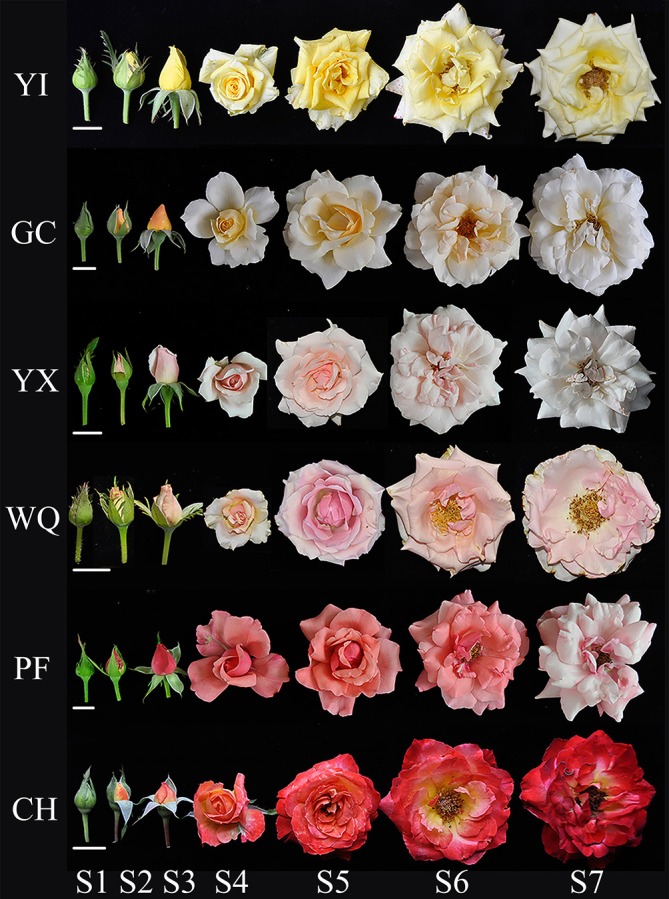
Flower phenotypes of six rose cultivars during flower development. Seven developing stages of rose flower: S1, unopened bud stage; S2, semi-opened stage; S3, fully-opened stage; S4, initial bloom stage; S5, full bloom stage; S6, bloomed stage; and S7, senescent stage. Bar = 2 cm.

The colors of all studied rose flowers turned darker from S1 to S5 before fading, except that of CH, whose color turned darker throughout development (Figure 1). To precisely evaluate the color of rose flowers, color parameters *L*^∗^, *a*^∗^, and *b*^∗^ of petals were measured. Significant differences were observed in color parameters among the six tested cultivars at different stages (*P* < 0.05, Table 2 and Supplementary Table S1). For cultivars GC, YX and PF, *L*^∗^ value decreased initiallyto reach the minimum at S2, S2, and S3, respectively, and later, was found to be increased as petals visually became paler. For cultivar YI, *L*^∗^ value increased from S1 to S3, and then decreased from S3 to S4, and finally increased from S4 to S7, with minimum and maximum value at S1 and S7, respectively. For cultivar CH, *L*^∗^ value decreased throughout the plant development as petals visually became darker. Parameter *a*^∗^ represents green and red from negative value to positive value. For yellow cultivars YI and GC, *a*^∗^ value increased to the maximum at S4 and S2, respectively, then decreased until S6 before increasing again. For pink cultivars YX and PF, *a*^∗^ value increased initially to reach the maximum at S2 and S3, respectively, and then decreased. For pink cultivar WQ, *a*^∗^ value showed unstable trend, with mimum and maximum value at S1 and S5, respectively. For orange cultivar CH, *a*^∗^ value increased throughout the flowering process. Parameter *b*^∗^ represents blue and yellow from negative value to positive value. For pink cultivars YX and PF, *b*^∗^ value decreased throughout the flowering period. For remaining four cultivars YI, GC, WQ and CH, *b*^∗^ value reached maximum at S4, S3, S2, and S4, respectively before declining. Two yellow rose cultivars, YI and GC both exhibited lower *a*^∗^ value and higher *b*^∗^ value. However, all pink rose cultivars showed higher *a*^∗^ value and lower *b*^∗^ value. The orange rose cultivars showed higher *a*^∗^ and *b*^∗^ value.

**Table 2:** Color parameters L^∗^, a^∗^, and b^∗^ on the adaxial surface of petals of six rose cultivars at different developing stages.

Stage	YI	GC	YX	WQ	PF	CH
***L*^∗^ on the adaxial surface of petals**						
S1	83.61 ± 1.14*d*^a^	85.16 ± 0.42*c*	78.31 ± 0.93*c*	83.57 ± 1.21*ab*	74.83 ± 4.94*b*	84.54 ± 1.28*a*
S2	85.27 ± 0.51*c*	78.44 ± 1.16*e*	77.04 ± 3.3*c*	77.03 ± 2.79*d*	63.09 ± 3.67*e*	76.03 ± 0.71*b*
S3	86.03 ± 0.39*c*	83.09 ± 2.6*d*	82.22 ± 1.7*b*	79.2 ± 1.92*cd*	55.55 ± 5.51*f*	74.7 ± 1.43*b*
S4	83.87 ± 0.11*d*	85.33 ± 0.38*c*	83.56 ± 0.12*b*	82.05 ± 1.30*bc*	62 1.04*e*	64.98 ± 0.99*c*
S5	85.37 ± 3.17*c*	87.01 ± 0.32*b*	84.26 ± 0.61*b*	72.26 ± 1.46*e*	65.43 ± 0.97*d*	61.30 ± 1.31*d*
S6	87.67 ± 0.51*b*	87.96 ± 0.23*b*	88.96 ± 0.17*a*	80.76 ± 0.75*bc*	72.22 ± 1.14*c*	59.02 ± 3.79*d*
S7	89.10 ± 0.39*a*	89.62 ± 3.16*a*	89.56 ± 0.23*a*	85.53 ± 0.19*a*	79.71 ± 0.92*a*	44.17 ± 2.81*e*
***a*^∗^ on the adaxial surface of petals**						
S1	-13.92 ± 0.27*e*	-9.49 ± 1.02*d*	-4.41 ± 2.05*e*	-11.67 ± 1.16*d*	3.75 ± 3.24*g*	-10.86 ± 0.63*e*
S2	-10.7 ± 2.12*d*	3.34 ± 2.17*a*	18.19 ± 2.7*a*	1.85 ± 0.69*c*	28 3.12*e*	11.58 ± 1.32*d*
S3	-9.21 ± 4.49*cd*	1.6 ± 1.38*a*	14.72 ± 7.98*b*	8.76 ± 3.35*b*	54.88 ± 5.33*a*	14.56 ± 4.07*d*
S4	-1.97 ± 0.08*a*	-2.22 ± 0.61*b*	9.84 ± 1.18*c*	3.39 ± 1.91*c*	51.14 ± 1.05*b*	42.23 ± 1.49*c*
S5	-5.97 ± 3.15*b*	-4.46 ± 4.42*c*	8.55 ± 0.6*c*	21.8 ± 2.33*a*	46.22 ± 0.61*c*	46.46 ± 2.26*bc*
S6	-8.32 ± 0.03*c*	-5.78 ± 0.68*c*	1.34 ± 1.20*d*	10.43 ± 1.04*b*	34.92 ± 3.67*d*	48.57 ± 4.78*b*
S7	-6.48 ± 0.36*b*	-2.33 ± 1.20*b*	-0.36 ± 0.23*d*	2.51 ± 0.32*c*	17.80 ± 5.25*f*	55.85 ± 4.90*a*
***b*^∗^ on the adaxial surface of petals**						
S1	48.64 ± 6.96*e*	31.01 ± 1.70*d*	32.22 ± 2.57*a*	34.45 ± 3.30*a*	30.78 ± 1.82*a*	40.33 ± 0.37*bc*
S2	56.1 ± 8.42*d*	43.74 ± 1.50*b*	20.99 ± 3.05*b*	37.38 ± 7.59*a*	29.21 ± 1.68*a*	40.6 ± 11.73*bc*
S3	59.68 ± 1.47*c*	53.13 ± 1.74*a*	13.53 ± 1.57*c*	34.98 ± 2.33*a*	26.37 ± 2.14*b*	41.41 ± 8.89*bc*
S4	78.42 ± 5.88*a*	34.37 ± 1.46*c*	10.3 ± 0.03*d*	27.29 ± 4.25*b*	23.23 ± 5.92*c*	54.82 ± 1.49*a*
S5	64.67 ± 1.58*b*	30.93 ± 1.22*d*	8.04 ± 0.63*de*	17.68 ± 0.73*c*	15.49 ± 4.97*d*	45.89 ± 4.00*ab*
S6	37.07 ± 0.53*f*	29.84 ± 0.62*d*	6.15 ± 0.43*e*	10.31 ± 0.44*d*	7.00 ± 3.22*e*	36.37 ± 1.35*bc*
S7	22.70 ± 4.93 g	9.78 ± 0.89*e*	6.95 ± 0.93*e*	9.07 ± 1.33*d*	5.21 ± 2.06*e*	35.26 ± 3.02*c*


*^*a*^Each value presents the mean ± standard deviation (SD) of three independent replicates. Different letters represent significant (*P* < 0.05) differences between means according to analysis of variance (ANOVA) combined with duncan’s multiple range test.*

### Identification and Quantification of Anthocyanin

**FIGURE 2:**
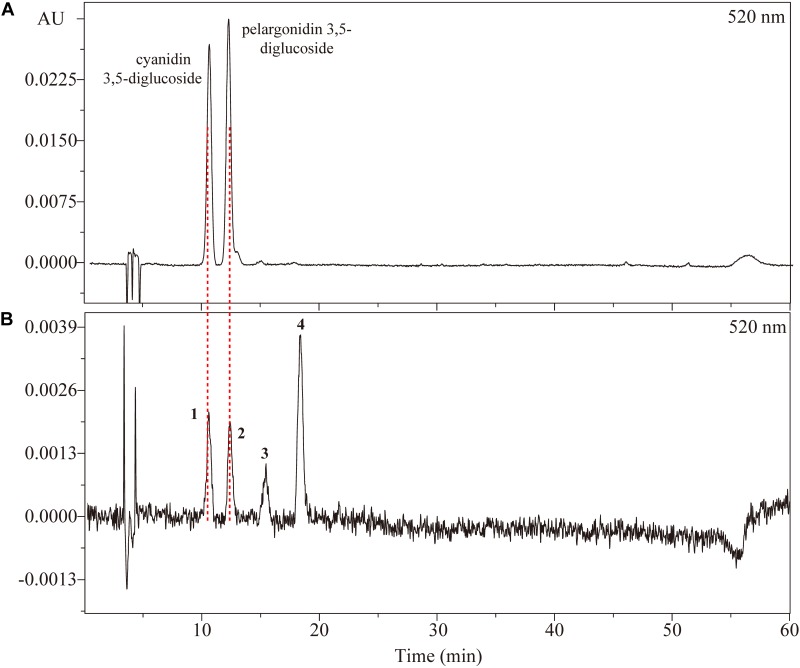
High performance liquid chromatography chromatogram of **(A)** a mix of standard anthocyanins and **(B)** anthocyanins extracted from petals of *Rosa* ‘Chacok’ (CH) at S5.

Analysis of anthocyanins was performed using HPLC-PDA-ESI-MS system. The chromatograms of anthocyanins extracted from rose petals of six cultivars are shown in Figure 2. Retention times, UV/Vis spectrum and MS are displayed in Table 3 and Supplementary Figures S1, S2.

**Table 3:** Chromatographic, spectroscopic, and mass spectral features of anthocyanin detected in petals of rose cultivar CH.

Peak	Identification	Rt (min)	λ_max_ (nm)^a^	%E_440_/E_vis-max_^b^	[M+H]^+^	ms/ms (*m*/*z*)
1	Cyanidin 3,5-diglucoside	10.48	277.2, 512.6	18.21	611.1	449.0, 287.0
2	Pelargonidin 3,5-diglucoside	12.13	276.0, 498.0	27.53	595.1	433.0, 271.1
3	Cyanidin 3-*O*-glucoside	14.88	279.6, 513.9	31.82	449.0	287.0
4	Pelargonidin 3-*O*-glucoside	17.79	277.2, 501.7	44.74	433.0	271.0


*^*a*^λ_*max*_ (nm) in the mobile phase, obtained by photodiode array detector (PDA). ^*b*^%E_*440*_/E_*vis-max*_ is the ratio of absorbance intensity at 440 nm to the visible maximum absorbance intensity.*

There were four types of anthocyanins detected in orange petals of CH (Figure 2). From MS data, peaks **1** and **3** represented identical fragment ions at *m/z* 287, indicating the presence of cyanidin aglycone. Similarly, peaks **2** and **4** were assumed to be derivatives of pelargonidin. The fragmention patterns of [M+H]^+^, [M+H-162]^+^ and [M+H-162-162]^+^ suggested that peaks **1** and **2** contained dihexose, while peaks **3** and **4** contained hexose. E_440_/E_vis-max_ values of four anthocyanins were 18.21, 27.53, 31.82, and 44.74%, respectively, indicating that both peaks **1** and **2** were glycosylated at positions 3 and 5, while peaks **3** and **4** were glycosylated at position 3. Thus, peaks **1**, **2**, **3,** and **4** were tentatively identified as cyanidin 3,5-diglucoside, pelargonidin 3,5-diglucoside, cyanidin 3-*O*-glucoside and pelargonidin 3-*O*-glucoside, respectively. Co-elution with anthocyanin standards (cyanidin 3,5-diglucoside chloride, pelargonidin 3,5-diglucoside chloride) also confirmed our assumption.

**FIGURE 3:**
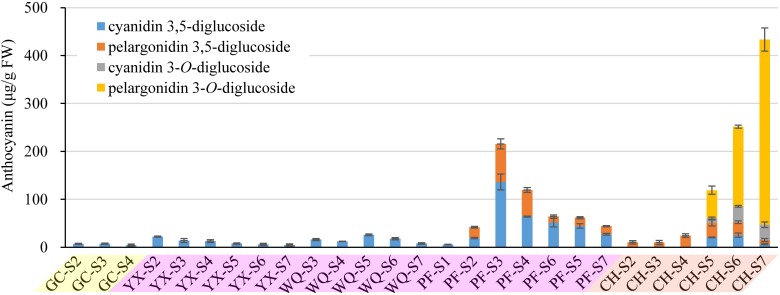
Contents of anthocyanins (detection at 520 nm) extracted from petals of rose cultivars at different developing stages. Data represent the means of three biological replicates ± SD. Because there was no anthocyanins detected in petals of YI throughout the flower development, so the graph present only five cuntivars.

The composition of anthocyanins exhibited substantial differences in petals of six rose cultivars at different blooming stages. The content of individual anthocyanin showed varied trends. For yellow cultivars YI and GC, there was no or very low anthocyanin content in petals during different stages. Maximum anthocyanin content was found to be ∼7 μg/g in the petals of GC at S3. For pink cultivar PF, cyanidin 3,5-diglucoside and pelargonidin 3,5-diglucoside were detected in petals at S1-7 and S2-7, respectively. Total anthocyanin content reached maximum at S3 (215.59 μg/g, FW), and minimum at S1 (5.54 μg/g, FW). Forpink cultivars YX and WQ, only one kind of anthocyanin (cyanidin 3,5-diglucoside) was detected at S2-7 and S3-7, respectively. The content of cyanidin 3,5-diglucoside in petals of YX and WQ was found to be maximum at S2 (22.13 μg/g, FW) and S5 (25.89 μg/g, FW), respectively. No anthocyanins were detected in petals of YX at S1 and WQ at S1-2. For orange cultivar CH, all identified anthocyanins were detected in petals at S5-7. Petals at S2-4 contained only one kind of anthocyanin (pelargonidin 3,5-diglucoside). No anthocyanin was detected in petals at S1. The content of cyanidin 3,5-diglucoside and cyanidin 3-*O*-glucoside both increased first and then decreased from S5 to S7. However, the content of pelargonidin 3,5-diglucoside decreased from S2 to S3, and increased from S3 to S5, before decreasing from S5 to S7. The concentration of total anthocyanins increased throughout the flower development, reaching the highest level at S7 (433.39 μg/g, FW) (Figure 3).

In this study, cyanidin 3,5-diglucoside was detected in the petals of five rose cultivars, such as GC, YX, WQ, PF and CH, and pelargonidin 3,5-diglucoside was detected in the petals of two rose cultivars, such as PF and CH. The content of cyanidin/pelargonidin 3,5-diglucoside accounted for 100.00% of total anthocyanins in all tested samples except the petals of CH at S5-7. Both cyanidin 3-*O*-glucoside and pelargonidin 3-*O*-glucoside were detected only in the petals of cultivar CH. In contrast, the petals of orange cultivar CH showed highest total anthocyanins content, followed by pink cultivars PF, WQ, YX, and yellow cultivars GC and YI (Supplementary Table S4).

### Flavonol Profiles in Rose Petals

**FIGURE 4:**
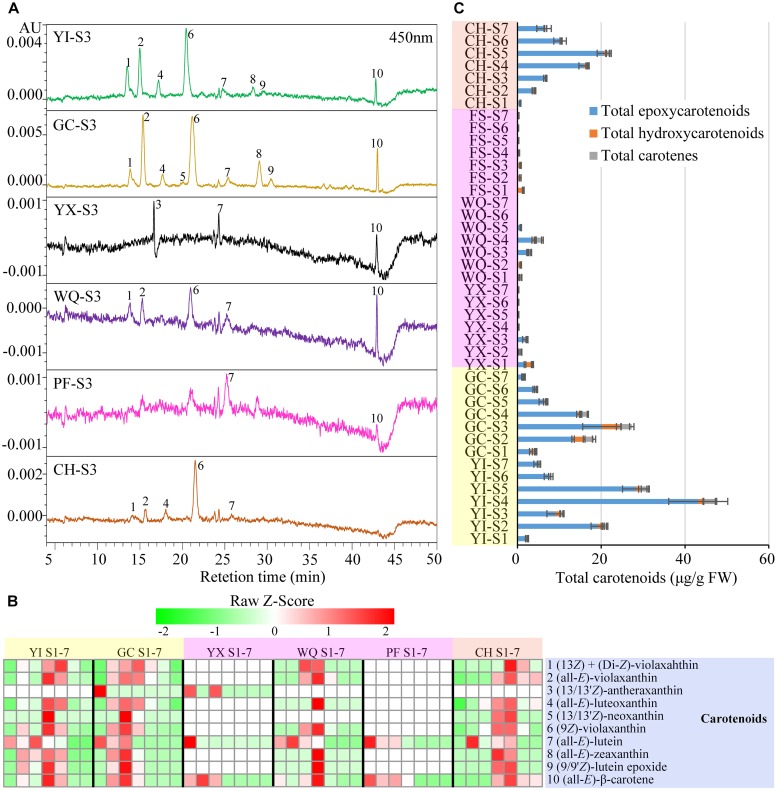
Contents of flavonols extracted from rose petals of six cultivars at different developing stages. **(A)** HPLC chromatograms of flavonols (detection at 350 nm): **1**, kaempferol 3-*O*-rhamnoside-7-*O*-glucoside; **2**, quercetin 3-*O*-glycoside; **3**, quercetin 7-*O*-glucoside; **4**, flavan-3-ol derivative; **5**, kaempferol 3-*O*-rutinoside; **6**, kaempferol 3-*O*-glucoside; **7**, kaempferol 3-*O*-glucuronide; **8**, kaempferol 3-*O*-(galloyl)-glucoside; **9**, quercetin 7-*O*-rhamnoside; **10**, kaempferol 3-*O*-xyloside; **11**, kaempferol 7-*O*-glucoside; **12**, kaempferol 3-*O*-arabinoside; **13**, kaempferol 3-*O*-hexoside; **14**, kaempferol 3-*O*-rhamnoside; **15**, kaempferol 3-*O*-glycoside 1; **16**, kaempferol 3-*O*-glycoside 2; **17**, kaempferol 7-*O*-(galloyl)-glucoside; **18**, kaempferol 3-*O*-glycoside 3; **19**, kaempferol 3-(*p-*coumaroyl)-glucoside; **20**, kaempferol. **(B)** A heat map of the individual flavonol contents. Row represents Z-Score normalization of the concentation of identified flavonols and column represents tested samples. Cells are colored based on concentrations in rose petals. Red represents relatively high concentration and green represents relatively low concentration of the identified flavonols in petals of each rose cultivar at seven developing stages. Each value represents the average of three biological replicates. **(C)** The concentration of total flavonols. Data represent the means of three biological replicates ± SD.

A total of 20 flavonols were detected in the petals of all tested cultivars by HPLC-PDA analysis at 350 nm. They were identified by comparing to their corresponding standards, elution order, λ_max_ and MS as described in our previous paper (Wan et al., 2018) (Supplementary Table S4). The isomers were identified by the elution order or λ_max_. For glycosylated flavonols, elution order is 3-*O*-rutinoside, 3-*O*-glucoside, 3-*O*-xyloside, 3-*O*-arabinoside and 3-*O*-rhamnoside (Schieber et al., 2001). The λ_max_ of 3-*O*-glycosides is shorter than that of 7-*O*-glycosides by ∼12–17 nm (Singh et al., 2010). Compounds were tentatively identified as **1** kaempferol 3-*O*-rhamnoside-7-*O*-glucoside (El-Desoky et al., 2001), **2** quercetin 3-*O*-glycoside, **3** quercetin 7-*O*-glucoside, **4** flavan-3-ol derivative, **5** kaempferol 3-*O*-rutinoside (Baek et al., 1994), **6** kaempferol 3-*O*-glucoside (Xiao et al., 2006), **7** kaempferol 3-*O*-glucuronide, **8** kaempferol 3-*O*-(galloyl)-glucoside (Sarangowa et al., 2013), **9** quercetin 7-*O*-rhamnoside, **10** kaempferol 3-*O*-xyloside, **11** kaempferol 7-*O*-glucoside (Xu et al., 2008), **12** kaempferol 3-*O*-arabinoside (Xiao et al., 2006), **13** kaempferol 3-*O*-hexoside, **14** kaempferol 3-*O*-rhamnoside (Zhizhen et al., 2003), **15** kaempferol 3-*O*-glycoside 1, **16** kaempferol 3-*O*-glycoside 2, **17** kaempferol 7-*O*-(galloyl)-glucoside, **18** kaempferol 3-*O*-glycoside 3, **19** kaempferol 3-(*p*-coumaroyl)-glucoside, and **20** kaempferol (Xiao et al., 2006), respectively. HPLC chromatograms of flavonols extracted from tested samples are shown in Figure 4A. Significant differences were observed in the contents of flavonols among different cultivars at seven developing stages. In order to visualize these differences, individual flavonol contents were normalized by Z-Score and expressed as heat map (Figure 4B). The concentration of total flavonols is shown in Figure 4C.

In yellow cultivars, 18 and 14 flavonols were detected in the petals of YI and GC, respectively. In the petals of YI, two major flavonols were kaempferol 3-*O*-rhamnoside (85.53–460.02 μg/g FW) and kaempferol 7-*O*-glucoside (54.89–291.12 μg/g FW). The content of most flavonols increased initially and reached the maximum at S2 or S3 before decresing. The content of total quercetins, total kaempferols and total flavonols exhibited similar profiles, with maximum levels observed at S2, at 53.58, 1050.97, and 1104.54 μg/g FW, respectively, and minimum levels at S7, S1, and S1, respectively. Further, in the petals of GC, kaempferol 3-*O*-rhamnoside and kaempferol 3-*O*-glucoside were two main flavonols at 144.89–525.81 μg/g FW and 40.90–177.04 μg/g FW, respectively. The concentration of almost all flavonols increased initially and reached the maximum at S2 before decreasing. The content of total quercetins, total kaempferols and total flavonols similar trend as that of individual flavonols, with maximum level at S2 (11.17, 1002.73, and 1013.90 μg/g FW, respectively), and minimum at S6 (1.20, 328.23, and 329.43 μg/g FW, respectively).

In pink cultivars, 14, 18, and 16 flavonols were detected in the petals of YX, WQ, and PF, respectively. Maximum content of kaempferol 3-*O*-rhamnoside was observed in the petals of these three cultivars, followed by kaempferol 3-*O*-glucoside, kaempferol 7-*O*-glucoside, and kaempferol 3-*O*-glucoside, respectively. In petals of YX, the concentration of individual flavonols exhibited different trends during blooming period. Interestingly, kaempferol 3-*O*-arabinoside was detected only at S7 (2.37 μg/g FW). The content of quercetin 7-*O*-glucoside, flavan-3-ol derivative, kaempferol 3-*O*-rutinoside, and total quercetins were found to be maximum at S1, and gradually reduced during flowering period. The contents of remaining 10 flavonols, total kaempferols and total flavonols increased first and reached the maximum levels at S2, before decreasing. Highest content of total kaempferols and total flavonols were found to be 1616.14 and 1634.83 μg/g FW, respectively. In WQ petals, the content of different individual flavonols varied greatly during blossoming period. The concentration of quercetin 3-*O*-glycoside was found to be maximum at S1 (12.00 μg/g FW) and minimum at S4 (3.70 μg/g FW), and then increased from S4 to S7. The concentration of quercetin 7-*O*-rhamnoside, kaempferol 7-*O*-glucoside, kaempferol 3-*O*-glycoside 1, kaempferol 7-*O*-(galloyl)-glucoside, and total quercetins showed variable trend during flowering period, and was lowest at S1 with maximum value at S6, S3, S6, S3, and S3, respectively. The concentration of remaining 13 individual flavonols, total kaempferols, and total flavonols exhibited variable trend showing highest/lowest levels at different stages. Maximum values of total kaempferols content and total flavonols content were detected in the petals at S2 at 1378.42, and 1444.57 μg/g FW, respectively. In PF petals, the content of individual flavonols exhibited variable trends. The concentration of many individual flavonols, such as peaks **1**, **4**, **5**, **8**, **10**, **13**, **14**, **15** and **20**, shared similar variations, with the maximum levels at S3 and minimum levels at S1. The content of peaks **2** and **6** also showed a variable trend, with maximum level at S2 and S3, respectively. The concentration of peak **3** showed highest level at S1 at 23.75 μg/g FW, before gradually decreasing. The content of peaks **11**, **18**, and **19** showed highest levels at S4, S3, and S3, respectively. Total quercetin content decreased first and then increased, showing the highest and lowest level at S1 (43.92 μg/g FW) and S6 (7.14 μg/g FW), respectively. The concentration of total kaempferols (529.73 μg/g FW) and total flavonols (573.65 μg/g FW) was minimum at S1, and reached maximum at S3 (2352.71 and 2385.92 μg/g FW, respectively), showing decreasing trend from S3 to S6, and increasing trend from S6 to S7.

In orange cultivar CH, 17 flavonols were detected in the petals. Two dominant flavonols were kaempferol 3-*O*-rhamnoside (98.60–218.66 μg/g FW) and kaempferol 7-*O*-glucoside (28.41–133.46 μg/g FW). The content of peaks **1**, **2**, **3**, **4**, **8**, **10**, **13**, **14**, **15**, **16**, **17**, and **20** demonstrated different changing trends, with maximum and minimum level at different stages. The concentration of peaks **5**, **6**, and **7** decreased first and then increased with maximum value observed at S1, S1, and S7, respectively, and minimum value at S5, S4, and S4, respectively. The concentration of peak **11** (kaempferol 7-*O*-glucoside) increased first and then decreased, with maximum value at S3 (218.66 μg/g FW), and minimum at S1 (98.60 μg/g FW). Total quercetins content, total kaempferols and flavonols varied greatly during the flowering period, with maximum value at S3 (39.33, 560.47, and 599.80 μg/g FW, respectively), and minimum at S4 (12.83 μg/g FW), S1 (291.20 μg/g FW), and S1 (323.99 μg/g FW), respectively.

In the petals of six rose cultivars, peak **14** (kaempferol 3-*O*-rhamnoside) was uniformly the most abundant individual flavonol, the content of which was higher than 26.95 μg/g FW in all tested samples, accounting for 8.77–62.30% of total flavonols. Total kaempferol content was much higher than total quercetin content, accounting for 75.03–99.64% of total flavonols. On comparison, the average of total flavonols content was maximum in the petals of pink cultivar PF (1203.17 μg/g FW), followed by other pink cultivars WQ and YX and yellow cultivars YI and GC, with minimum content in the petals of orange cultivar CH (443.10 μg/g FW) (Supplementary Table S5).

### Carotenoid Profiles in Rose Petals

**FIGURE 5:**
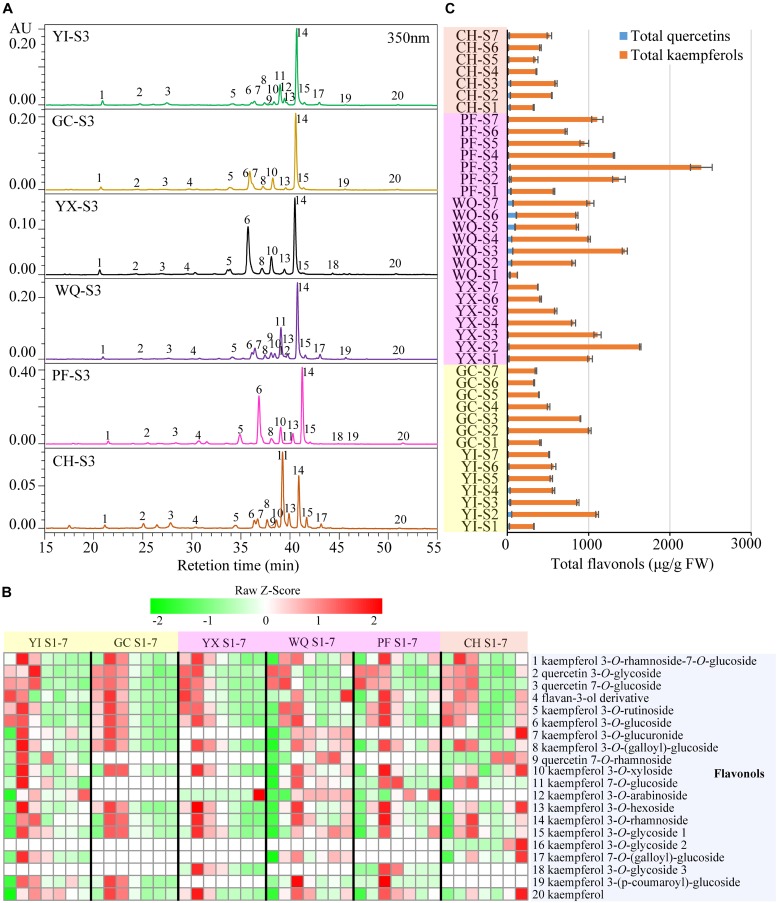
Contents of carotenoids extracted from rose petals of six cultivars at different developing stages. **(A)** HPLC chromatograms of carotenoids (detection at 450 nm): **1**, (13*Z*) + (di-*Z*)-violaxanthin; **2**, (all-*E*)-violaxanthin; **3**, (13/13′*Z*)-antheraxanthin; **4**, (all-*E*)-luteoxanthin; **5**, (13/13′*Z*)-neoxanthin; **6**, (9*Z*)-violaxanthin; **7**, (all-*E*)-lutein; **8**, (all-*E*)-zeaxanthin; **9**, (9/9′*Z*)-lutein epoxide; **10**, (all-*E*)-β-carotene. **(B)** A heat map of the individual carotenoid contents. Row represents Z-Score normalization of the concentation of identified carotenoids and column represents tested samples. Cells are colored based on concentrations in rose petals. Red represents relatively high concentration and green represents relatively low concentration of the identified carotenoids in petals of each rose cultivar at seven developing stages. Each value represents the average of three biological replicates. **(C)** The concentration of total carotenoids. Data represent the means of three biological replicates ± SD.

A total of 10 carotenoids were detected in the petals of six rose cultivars by HPLC-PDA analysis. These 10 carotenoids were identified as **1** (13*Z*) + (di-*Z*)-violaxanthin, **2** (all-*E*)-violaxanthin, **3** (13/13′*Z*)-antheraxanthin, **4** (all-*E*)-luteoxanthin, **5** (13/13′*Z*)-neoxanthin, **6** (9*Z*)-violaxanthin, **7** (all-*E*)-lutein, **8** (all-*E*)-zeaxanthin, **9** (9/9′*Z*)-lutein epoxide, and **10** (all-*E*)-β-carotene according to our previous study (Wan et al., 2018) (Supplementary Table S3). HPLC chromatograms of carotenoids, heat map of individual carotenoid contents and total carotenoids contents are shown in Figure 5.

In yellow cultivars, 9 and 10 carotenoids were detected in the petals of YI and GC, respectively. Peak **6** (9*Z*)-violaxanthin was the most predominant carotenoid in the petals of YI (0.93–24.01 μg/g FW) and GC (0.40–11.06 μg/g FW). In YI petals, the contents of individual carotenoids exhibited different changing trends, such as peak **1** (13*Z*) + (di-*Z*)-violaxanthin, **2** (all-*E*)-violaxanthin, **4** (all-*E*)-luteoxanthin, **6** (9*Z*)-violaxanthin, **8** (all-*E*)-zeaxanthin, **9** (9/9′*Z*)-lutein epoxide, and **10** (all-*E*)-β-carotene. Peak **1** (13*Z*) + (di-*Z*)-violaxanthin showed maximum content at S5, and peak **7** (all-*E*)-lutein showed maximum content at S3, while remaining carotenoids reached maximum levels at S4. Total carotenoids content was highest at S4 (47.20 μg/g FW), and was lowest at S1 (2.65 μg/g FW). In GC petals, the contents of individual carotenoids and total carotenoids increased first, showing maximum level at S3, and then decreased gradually from S3 to S7.

In pink cultivars, only three carotenoids were detected in YX petals from S1 to S3, with no detection from S4 to S7. Total carotenoids content was found to be maximum at S1 (3.80 μg/g FW). In WQ petals, WQ, eight carotenoids were detected from S1 to S5, with no detection from S6 to S7. Two major carotenoids were found to be peaks **6** (9*Z*)-violaxanthin (0.00–1.98 μg/g FW) and **2** (all-*E*)-violaxanthin (0.00–1.16 μg/g FW). Peak **1** (13*Z*) + (di-*Z*)-violaxanthin exhibited maximum level at S3, while peak **7** (all-*E*)-lutein showed highest content at S2, and remaining six carotenoids peaked at S4. Total carotenoids content increased initially, reaching the highest level at S4 (5.95 μg/g FW), and then decreased. In PF petals, only two carotenoids were detected from S1 to S4 [peak **7** (all-*E*)-lutein and peak **10** (all-*E*)-β-carotene]. The contents of peaks **7** and **10** and total carotenoids were highest at S1 (1.28, 0.30, and 1.58 μg/g FW, respectively), and then decreased throughout the flowering period.

In orange cultivar CH, 9 carotenoids were detected in the petals from S1 to S7. Two major carotenoids were (9*Z*)-violaxanthin (0.42–10.34 μg/g FW) and (all-*E*)-violaxanthin (0.27–5.24 μg/g FW). The contents of the majority of detected carotenoids increased first, reaching the highest level at S5, and then decreased. Total carotenoids content was minimum at S1 (0.70 μg/g FW), and increased from S1 to S5 to reach the maximum level at S5 (21.64 μg/g FW), and then decreased from S5 to S7.

Average total carotenoids content was maximum in the petals of yellow cultivars YI (18.04 μg/g FW) and GC (11.21 μg/g FW), followed by orange cultivar CH (9.64 μg/g FW) and was found to be minimum in the petals pink cultivars WQ, YX, and PF. In the petals of yellow and orange rose cultivars, peak **6** (9*Z*)-violaxanthin was found to be the most dominant individual carotenoid. Moreover, the content of total epoxycarotenoids was much higher than any other carotenoids, accounting for 70.60–100.00% of total carotenoids (Supplementary Table S6).

### Contributions of Flavonoids and Carotenoids to Various Colors of Rose

**FIGURE 6:**
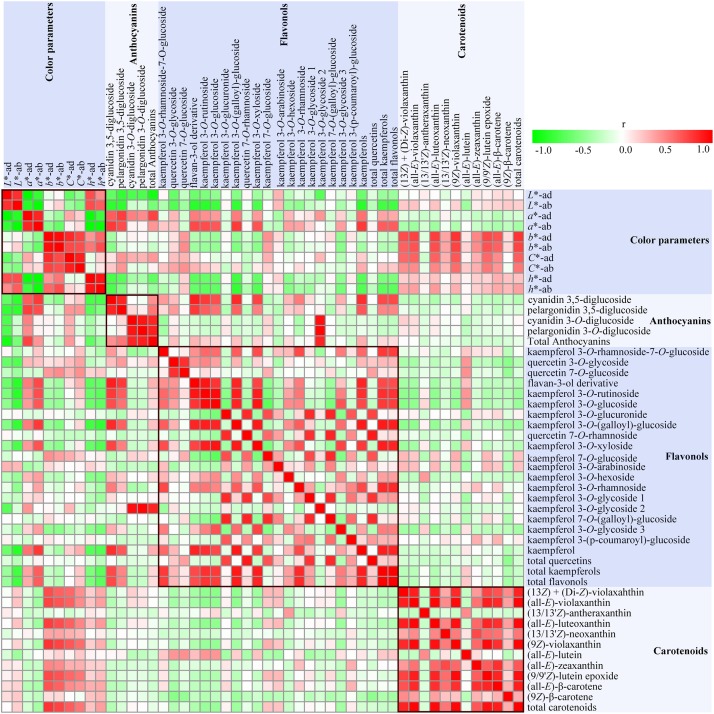
A heat map of correlation matrix of color parameters and 47 compounds from petals of six rose cultivars at seven developing stages. Each square indicates Pearson’s correlation coefficient for a pair of data, and the intensity of red and gree colors in the heat map indicates the level of positive and negative correlation, respectively.

In order to analyze the relationship between flower color and pigment contents in rose petals, Pearson’s correlation coefficients were calculated among color parameters, anthocyanins content, flavonols content, and carotenoids content and displayed as a heat map (Figure 6).

Strong correlations were observed among different color parameters and pigments content. On both sides of petals, *L*^∗^ values were negatively correlated with *a*^∗^ and *C*^∗^ (*P* < 0.05), and positively correlated with *h* (*P* < 0.01), with slight correlation with *b*^∗^. Most of the individual anthocyanins content and total anthocyanins content demonstrated significant positive correlations with *a*^∗^ and *C*^∗^, and negative correlations with *L*^∗^ and *h* (*P* < 0.05), with no apparent correlations with *b*^∗^. The absolute values of correlation coefficients between anthocyanins content and *L*^∗^ and *a*^∗^ on adaxial sides of petals were more than 0.47 (*P* < 0.01). On comparison, anthocyanidin 3,5-diglucoside exhibited stronger correlation with color parameters than anthocyanin 3-*O*-glucoside. Both *L*^∗^ and *h* values showed significant negative correlations with the contents of 8–9 individual flavonols, total kaempferols, and total flavonols. Conversely, *a*^∗^ was found to be significantly positively correlated with the contents of nine individual flavonols, total kaempferols, and total flavonols. However, there were no obvious correlations between all color parameters and total quercetins content, and between *b*^∗^ and all flavonol contents. Majority of individual carotenoids content and total carotenoids content were positively correlated with *h*, and most significantly positively correlated with *b*^∗^ and *C*^∗^, with least correlated with *L*^∗^ and *a*^∗^.

The correlations among flavonol, anthocyanin, and carotenoid metabolism were also evaluated. Total anthocyanins content showed significant and positive correlation to the concentration of all four identified anthocyanins (*P* < 0.01). Most significant positive correlations were observed between cyanidin 3,5-diglucoside and pelargonidin 3,5-diglucoside, and cyanidin 3-*O*-glucoside, and pelargonidin 3-*O*-glucoside (*P* < 0.01). However, no significant correlations were observed between cyanidin/pelargonidin 3,5-diglucoside and cyanidin/pelargonidin 3-*O*-glucoside. Most of individual flavonol contents, total kaempferols content, and total flavonols content showed significant positive correlations with cyanidin/pelargonidin 3,5-diglucoside content (*P* < 0.05), with no significant correlation with cyanidin/pelargonidin 3-*O*-glucoside. There were significant positive correlations among the contents of most of individual flavonols, total kaempferols, and total flavonols (*P* < 0.05). Further, except for (13/13′*Z*)-antheraxanthin and (all-*E*)-lutein, significant positive correlations were observed among eight individual carotenoids content and total carotenoids content (*P* < 0.01). However, no obvious correlations were observed between carotenoid content and anthocyanin content and flavonol content.

Correlation analysis showed that there were many pigments influencing the rose flower color. Thus, MLR analysis was further performed to estimate the type of pigment that greatly affected the rose flower color. Color parameters *L*^∗^, *a*^∗^, and *b*^∗^ were selected as dependent variables, and concentration of 40 pigments were chosen as independent variables. Significant statistical results obtained are as follows:

$$L*=82.253-0.317\text{pelargonidin3,5-diglucoside-0.045TA}\left(R^{2}=0.660,P=2.766E-10\right)$$

$$a*=4.418+0.907\text{pelargonidin3,5-diglucoside}\left(R^{2}=0.582,P=9.168E-10\right)$$

$$b*=21.177+2.755(9Z)-violaxanthin\left(R^{2}=0.660,P=1.490E-9\right)$$

Multiple linear regression analysis indicated that pelargonidin 3,5-diglucoside was the major factor that affected the values of *L*^∗^ and *a*^∗^, with negative effects on *L*^∗^ value, but positive effects on *a*^∗^ value. TA (total anthocyanins) was the other important factor that exhibited negative effects on *L*^∗^ value. Further, (9*Z*)-violaxanthin was found to be the primary factor that positively influenced *b*^∗^ value.

## Discussion

### Anthocyanins Are the Key Pigments in Pink and Orange Petals of Rose

Anthocyanins are important hydrophilic pigments in many plant tissues. Structural information provided by LC–MS, in combination with UV/Vis spectrum is a very useful method for the identification of anthocyanins. Harborne (1958) found that E_440_/E_vis-max_ values of anthocyanidin, glycosylated at position 3 was much higher than corresponding anthocyanidin, glycosylated at positions 3 and 5. Considering that anthocyanidin 3-glycoside existed abundantly in the petals of modern rose cultivars (Biolley et al., 1994a; Mikanagi et al., 2000; Schmitzer et al., 2009), anthocyanins **3** and **4** were tentatively assumed as cyanidin 3-*O*-glucoside and pelargonidin 3-*O*-glucoside.

Using commercially available standards, this study identified four different types of anthocyanins in the petals of six rose cultivars during seven developing stages. Among them, cyanidin 3,5-diglucoside, and pelargonidin 3,5-diglucoside were the two major anthocyanins in most of the tested samples (Figure 2). High content of anthocyanidin 3,5-diglucoside has been previously reported in the petals of genus *Rosa* (Raymond et al., 1995; Mikanagi et al., 2000; Schmitzer et al., 2009, 2010, 2012; Lee et al., 2011). This can be attributed to one particular anthocyanidin glucosyltransferase in rose that is always glycosylated at two different positions on aglycone (Ogata et al., 2005).

Previous studies reported strong correlations between anthocyanin concentration and color parameters (Schmitzer et al., 2009, 2010). In this study, considerable amounts of anthocyanins were detected in pink and orange petals of rose cultivars, but not in yellow rose cultivars. Correlation analysis showed that there are many kinds of pigments that influences rose flower color. Moreover, MLR analysis confirmed that pelargonidin 3,5-diglucoside and TA are the essential factors responsible for rose color. With the increase in pelargonidin 3,5-diglucoside content, *a*^∗^ value increased and *L*^∗^ value decreased, indicating the intense red color of the petals. Thus, it could be speculated that pink color of rose petals are due to anthocyanins content.

During flower development, anthocyanins content increased first and then decreased in the petals of rose cultivars YI, GC, YX, WQ, and PF. Similar phenomena were observed in the petals of various plants, such as rose cultivar ‘KORcrisett’ (Schmitzer et al., 2009), eight groundcover rose cultivars (Schmitzer et al., 2010) and three crabapple cultivars (Zhang et al., 2017). The decrease in anthocyanins content could be attributed to the expansion of petal cells and degradation of anthocyanins (Luo et al., 2017). However, the concentration of cyanidin 3-*O*-glucoside, pelargonidin 3-*O*-glucoside and TA increased in the petals of CH throughout the flower development. As a result, the flower color of CH turned darker in the progress of flower development. This can be significant in solving the problem of quick discoloration of many flowering plants. Therefore, CH could be an important germplasm for cultivating non-fading colors in modern rose cultivars.

### Flavonols Are Accessory Pigments in the Petals of Different Colors in Rose

In the present study, flavonols were detected in all yellow, pink and orange petals of rose, among which CH exhibited the lowest content of TF (Figure 4 and Supplementary Table S5). With respect to the highest content of TA in CH petals, it could be considered that there is competition between flavonols and anthocyanins for common substrate. This assumption was supported by many previous studies that showed that flavonols play crucial role in the pigmentation of flowers by competing with anthocyanin for dihydroflavonol (Czemmel et al., 2009; Tian et al., 2015; Luo et al., 2016). Sarangowa et al. (2014) reported that quercetin 3-*O*-glucoside (**2**) and kaempferol 3-*O*-glucoside (**6**) were the main flavonols in the petals of five *R*. *gallica* cultivars and three *R*. *damascene* cultivars. On the contrary, results of this study indicated that kaempferol 3-*O*-rhamnoside (**14**) was the major flavonol in the petals of all tested rose cultivars. This showed that there are significant differences in major flavonol glycosides among different rose species and cultivars.

Many previous studies have reported that aglycones of flavonols were kaempferol and quercetin in the petals of rose species and cultivars (Cai et al., 2005; Kumar et al., 2009; Sarangowa et al., 2014). In this study, both of these aglycones were detected in the petals of six rose cultivars, but the content of kaempferol derivatives was much higher than that of quercetin derivatives. Similar results were observed in the petals of 50 *R. hybrida* cultivars (Biolley et al., 1994b) and yellow rose cultivar ‘Sun City’ (Wan et al., 2018). Considering the pharmacological activities of kaemferol derivatives, petals of rose cultivars, especially PF that showed maximum content of total flavonols (573.65–2385.92 μg/g FW, Supplementary Table S5), could be the potential resource for kaempferols.

In addition, the correlation analysis showed that contents of most flavonols, such as TK and TF were positively correlated with the values of *L*^∗^ and *h*, and negatively correlated with the values of *a*^∗^. However, MLR analysis indicated that flavonols were not the major influencing factors of color parameters *L*^∗^, *a*^∗^, and *b*^∗^ (Li et al., 2015). These results suggested that flavonols might play roles in the pigmentation of flowers through co-pigmentation with anthocyanin. Gordillo et al. (2015) reported that co-pigmentation with flavonols could make anthocyanins more stable.

### Carotenoids Are the Key Pigment in the Yellow and Orange Petals of Rose

In this study, 10 carotenoids were detected in the petals of all tested rose cultivars, with the highest content in yellow petals, followed by orange petals and pink petals (lowest) (Figure 5 and Supplementary Table S6). This result is in agreement with that of Eugster and Märki-Fischer (1991), who reported that yellow colors were contributed by carotenoids and orange colors were produced by the mixture of carotenoids and anthocyanins. According to correlation and MLR analysis, it was observed that carotenoids, especially (9*Z*)-violaxanthin were the key factors that influence the values of *b*^∗^. Thus, it can be concluded that carotenoids are the key pigments responsible for the yellow color of rose.

In addition, carotenoids have huge application potential in food, medicine and cosmetic industries due to their antioxidant capacity (Arathi et al., 2015; Di Pietro et al., 2016). In this study, (9*Z*)-violaxanthin was the major carotenoid in yellow and orange petals in most developing stages. The content of (9*Z*)-violaxanthin was highest in YI petals at S4 (24.01 μg/g, FW), however, it was lower than that in the petals of ‘Sun City’ at S4 (142.71 μg/g, FW) (Wan et al., 2018). Since, it was higher than that of natural sources (>20 μg/g) (Britton and Frederick, 2009), the petals of yellow rose cultivar YI could be a valuable source for carotenoid extraction.

## Conclusion

This study provided a systematic report on the composition of hydrophilic and lipophilic pigments in the petals of two yellow rose cultivars, three pink cultivars and one orange cultivar at seven developing stages. Four anthocyanins, 20 flavonols, and 10 carotenoids were detected in the rose petals. Qualitative and quantitative analysis showed that pink colors were mainly imparted by cyanidin/pelargonidin 3,5-diglucoside and cyanidin/pelargonidin 3-*O*-glucoside, and yellow colors were endowed by (all-*E*)-violaxanthin, (9*Z*)-violaxanthin, and (all-*E*)-β-carotene. Moreover, orange colors were imparted by a combination of these pigments. Correlation and MLR analysis indicated that pelargonidin 3,5-diglucoside, total anthocyanins and (9*Z*)-violaxanthin were the major factors that affected the colors of rose petals. In addition, petals of orange cultivar CH, pink cultivar PF and yellow cultivar YI were found to possess high content of pelargonidin 3-*O*-glucoside, kaempferol 3-*O*-rhamnoside, and (9*Z*)-violaxanthin, respectively, which have been proved to have numerous biological activities. This study could provide a theoretical basis for rose breeding to achieve specific flower color. In addition, it would also be conducive to develop rose petals as a natural source of anthocyanins, flavonols, and carotenoids.

## Author Contributions

HW and CY performed most of the experiments, data analysis, and wrote the first draft. XG assisted HW to take care of the plant materials and collect the tested samples. YH, LL, TZ, and JW revised and critically evaluated the manuscript. QZ, TC, and HP helped in the planning, mentoring and supervision of the experiments, data interpretation and manuscript preparation. All the authors approved the final version of the manuscript.

## Conflict of Interest Statement

The authors declare that the research was conducted in the absence of any commercial or financial relationships that could be construed as a potential conflict of interest.

## Abbreviations

APCI: atmospheric pressure chemical ionization source
ESI: electrospray ionization
HPLC: high performance liquid chromatography
MS: mass spectrometry
PDA: photodiode array detector
