# Supplementary material for: Determination of Flavonoids and Carotenoids and Their Contributions to Various Colors of Rose Cultivars (Rosa spp.)
Source: Front Plant Sci. 2019 Feb 12;10:123. doi: 10.3389/fpls.2019.00123 (PMC6379320; doi:10.3389/fpls.2019.00123)
Supplement: Supplementary file 1 [file Table_1.DOCX]

**Table S1 | Color parameters *L**, *a*∗ and *b** on the abaxial surface of petals of six rose cultivars at different developing stages**

| Stage | YI | GC | YX | WQ | PF | CH |
| --- | --- | --- | --- | --- | --- | --- |
| *L** on the abaxial surface of petals | | | | | | |
| S1 | 82.61 ± 0.65 f | 84.85 ± 3.69 d | 77.20 ± 2.18 c | 82.68 ± 2.29 a | 73.27 ± 2.31 b | 83.94 ± 2.65 a |
| S2 | 84.77 ± 0.40 e | 74.31 ± 3.47 f | 70.28 ± 4.32 d | 66.89 ± 1.28 e | 57.72 ± 2.46 e | 76.91 ± 1.74 bc |
| S3 | 86.12 ± 0.38 d | 80.93 ± 3.55 e | 77.66 ± 2.42 c | 75.82 ± 2.76 c | 49.83 ± 2.31 f | 78.20 ± 2.58 b |
| S4 | 84.66 ± 0.41 e | 85.86 ± 1.28 cd | 82.56 ± 2.18 b | 79.55 ± 2.76 b | 60.91 ± 2.39 d | 75.34 ± 2.62 bc |
| S5 | 87.38 ± 0.45 c | 87.13 ± 2.75 c | 82.32 ± 3.34 b | 70.63 ± 2.96 d | 60.47 ± 2.58 d | 71.40 ± 1.23 d |
| S6 | 89.76 ± 0.46 b | 89.04 ± 2.68 b | 90.09 ± 3.23 a | 77.74 ± 2.64 bc | 70.88 ± 1.12 c | 75.16 ± 2.23 c |
| S7 | 90.60 ± 0.47 a | 90.60 ± 2.94 a | 89.63 ± 3.83 a | 83.74 ± 2.77 a | 77.8 ± 2.97 a | 72.36 ± 2.20 d |
| *a** on the abaxial surface of petals | | | | | | |
| S1 | -14.63 ± 0.23 f | -10.26 ± 0.51 d | -1.23 ± 1.15 e | -11.32 ± 0.49 d | 5.40 ± 1.09 f | -11.44 ± 0.97 d |
| S2 | -9.29 ± 1.53 d | 9.04 ± 1.95 a | 29.61 ± 2.14 a | 19.50 ± 1.71 b | 39.74 ± 3.97 d | 8.38 ± 3.32 c |
| S3 | -10.95 ± 1.11 e | 6.91 ± 1.42 a | 26.6 ± 3.35 b | 17.6 ± 4.41 b | 60.92 ± 2.57 a | 4.30 ± 0.72 c |
| S4 | -2.89 ± 1.76 a | 0.11 ± 2.03 b | 16.53 ± 2.70 c | 12.27 ± 0.67 c | 54.55 ± 3.50 c | 17.21 ± 0.84 b |
| S5 | -5.97 ± 2.47 b | -1.99 ± 0.22 b | 14.79 ± 2.67 c | 30.06 ± 1.10 a | 57.01 ± 2.65 b | 25.44 ± 1.76 a |
| S6 | -7.43 ± 3.17 c | -4.99 ± 1.11 c | 2.14 ± 3.32 d | 19.67 ± 1.29 b | 39.39 ± 1.91 d | 18.39 ± 5.42 b |
| S7 | -6.01 ± 2.34 b | -2.38 ± 0.51 b | 1.49 ± 3.09 d | 9.06 ± 1.29 c | 23.67 ± 2.34 e | 18.05 ± 1.74 b |
| *b** on the abaxial surface of petals | | | | | | |
| S1 | 49.63 ± 5.58 a | 34.72 ± 2.33 c | 36.08 ± 1.47 a | 34.35 ± 3.68 a | 33.88 ± 1.13 a | 43.45 ± 1.38 b |
| S2 | 61.44 ± 1.93 b | 40.95 ± 2.57 b | 15.85 ± 3.89 b | 27.75 ± 4.54 b | 28.08 ± 0.55 b | 42.51 ± 4.06 b |
| S3 | 52.2 ± 3.18 c | 51.16 ± 1.75 a | 8.72 ± 3.62 c | 27.11 ± 2.02 b | 16.95 ± 2.97 c | 47.57 ± 2.23 ab |
| S4 | 69.95 ± 3.6 cd | 36.21 ± 2.18 c | 6.61 ± 4.20 c | 20.2 ± 2.06 c | 12.27 ± 3.45 d | 50.29 ± 1.55 a |
| S5 | 54.45 ± 2.68 d | 28.85 ± 0.31 d | 2.41 ± 2.27 d | 7.53 ± 1.41 d | 7.91 ± 1.46 e | 43.05 ± 3.40 b |
| S6 | 32.88 ± 1.82 e | 29.4 ± 1.21 d | 6.97 ± 3.30 c | 4.62 ± 1.17 d | 1.66 ± 1.56 f | 29.48 ± 1.27 c |
| S7 | 21.00 ± 1.82 f | 10.35 ± 0.99 e | 5.62 ± 1.15 c | 6.69 ± 0.68 d | 2.37 ± 2.72 f | 20.93 ± 3.14 d |

*^a^* Each value presents the mean ± standard deviation (SD) of three independent replicates. Diﬀerent letters represent signiﬁcant (p < 0.05) diﬀerences between means according to analysis of variance (ANOVA) combined with duncan’s multiple range test.

Table S2 **|** Chromatographic, spectroscopic and mass spectrometric features of flavonoids detected in rose petals.

| peak | identifeication | Rt (min) | λ_max_ (nm) *^a^* | negative ion mode | | positive mode | |
| --- | --- | --- | --- | --- | --- | --- | --- |
|  |  |  |  | [M-H]^‑^ | MS/MS (*m/z*) | [M+H]^+^ | MS/MS (*m/z*) |
| **1** | kaempferol 3-*O*-rhamnoside-7-*O*-glucoside | 20.45 | 263.0, 342.7 | 593.1429 | 478.9149 | 595.1364 | 449.1014, 287.0474 |
| **2** | quercetin 3-*O*-glycoside | 24.69 | 253.6, 360.9 | nd | nd | nd | nd |
| **3** | quercetin 7-*O*-glucoside | 27.62 | 252.4, 365.1 | 463.0231 | 301.0233 | 465.0791 | 303.0158 |
| **4** | flavan-3-ol derivative | 29.95 | 266.6 | nd | nd | nd | nd |
| **5** | kaempferol 3-*O*-rutinoside | 34.15 | 264.2, 347.5 | 593.1418 | 447.0623, 285.0731 | 595.1724 | 449.1115, 287.0544 |
| **6** | kaempferol 3-*O*-glucoside | 36.17 | 264.2, 346.3 | 447.0839 | 285.0334 | 449.1237 | 287.0541 |
| **7** | kaempferol 3-*O*-glucuronide | 36.52 | 264.2, 346.3 | 461.0634 | 285.0312 | 463.0954 | 287.0569 |
| **8** | kaempferol 3-*O*-(galloyl)-glucoside | 37.62 | 265.4, 355.8 | 599.0939 | 447.0738, 285.0334 | 601.0146 | 449.1023, 287.0514 |
| **9** | quercetin 7-*O*-rhamnoside | 37.96 | 252.4, 362.9 | 447.0834 | 301.0229 | 449.1134 | 303.0148 |
| **10** | kaempferol 3-*O*-xyloside | 38.60 | 265.4, 347.5 | 417.0734 | 285.0139 | 419.0591 | 287.0563 |
| **11** | kaempferol 7-*O*-glucoside | 39.19 | 265.4, 318.8, 360.9 | 447.0837 | 285.0345 | 449.1134 | 287.0556 |
| **12** | kaempferol 3-*O*-arabinoside | 39.65 | 263.0, 342.7 | 417.0749 | 285.0518 | 419.1099 | 287.0583 |
| **13** | kaempferol 3-*O*-hexoside | 39.95 | 263.0, 346.3 | 447.0834 | 285.0353 | 449.1135 | 287.0568 |
| **14** | kaempferol 3-*O*-rhamnoside | 40.94 | 263.0, 342.7 | 431.0899 | 285.0282 | 433.1131 | 287.0528 |
| **15** | kaempferol 3-*O*-glycoside 1 | 41.79 | 265.4, 347.5 | nd | nd | nd | nd |
| **16** | kaempferol 3-*O*-glycoside 2 | 42.01 | 264.2, 347.5 | nd | nd | nd | nd |
| **17** | kaempferol 7-*O*-(galloyl)-glucoside | 43.30 | 266.6, 362.9 | 599.0947 | 447.0736, 285.0342 | nd | 287.0567 |
| **18** | kaempferol 3-*O*-glycoside 3 | 44.72 | 263.0, 342.7 | nd | nd | nd | nd |
| **19** | kaempferol 3-(p-coumaroyl)-glucoside | 46.57 | 265.4, 315.2 | 593.1067 | 447.0359, 285.0649 | 595.0951 | 449.1027, 287.0373 |
| **20** | kaempferol | 51.40 | 265.4, 365.1 | 285.0336 | nd | 287.0556 | nd |

*^a^* λ_max_ (nm) in the mobile phase (acidified water-acetonitrile), detected by photodiode array detector.

Table S3 **|** chromatographic, spectroscopic and mass spectrometric features of carotenoids detected in rose petals.

| peak | identifeication | Rt(min) | λ_max_(nm) *^a^* | %III/II *^b^* | %AB/AII *^c^* | [M+H]^+^(*m/z*) | ms/ms(*m/z*) |  |
| --- | --- | --- | --- | --- | --- | --- | --- | --- |
| **1** | (13*Z*) + (di-*Z*)-violaxanthin | 13.50 | 327.2, 410.2, 432.6, 456.8 | 45.47 | 27.35 | 601.4256 | 583.4147, 565.4057, 491.3510 |  |
| **2** | (all-*E*)-violaxanthin | 15.05 | 416.1, 438.6, 468.9 | 95.2 |  | 601.4252 | 583.4172, 565.4047, 491.3509 |  |
| **3** | (13/13'*Z*)-antheraxanthin | 17.08 | 329.1, 415.8, 438.7, 465.3 | 45.31 | 22.49 | 585.4389 | 567.4337, 549.4176 |  |
| **4** | (all-*E*)-luteoxanthin | 17.12 | 309.3, 397.6, 420.5, 447.1 | 98.1 |  | 601.4292 | 583.4163, 565.4049 |  |
| **5** | (13/13'*Z*)-neoxanthin | 19.24 | 329.2, 414.9, 438.6, 466.5 | 66.42 | 37.02 | 601.4247 | 583.4152, 565.4056 |  |
| **6** | (9*Z*)-violaxanthin | 20.22 | 326.0, 410.9, 433.8, 462.9 | 90.18 | 12.34 | 601.4251 | 583.4155, 565.4059, 509.5109, 491.3505 |  |
| **7** | (all-*E*)-lutein | 24.75 | 443.9, 472.6 | 67.29 |  | 569.4328 | 551.4222, 533.5638 |  |
| **8** | (all-*E*)-zeaxanthin | 28.18 | 423, 449.5, 477.4 | 37.41 |  | 569.4334 | 551.4237, 533.5626 |  |
| **9** | (9/9'*Z*)-lutein epoxide | 28.82 | 331.9, 416.2, 439.94, 467.7 | 65.35 | 10.24 | 585.4327 | 567.4351, 549.4179 |  |
| **10** | (all-E)-β-carotene | 41.93 | 425.1, 452.1, 478.6 | 30.81 |  | 537.4471 | 445.4434 |  |

*^a^* λ_max_ (nm) in the mobile phase (MTBE-methanol-water), detected by photodiode array detector.

*^b^* %III/II is the ratio of the peak height of band III to that of band II.

*^c^* %AB/AII is the ratio of the height of the *cis*-peak band to that of band II.

**Table S4 | Statistics analysis of total anthocyanins content in petals of six rose cultivars**

|  | Average μg/g FW | Standard deviation | Minimum μg/g FW | Maximum μg/g FW |
| --- | --- | --- | --- | --- |
| YI | - | - | - | - |
| GC | 2.73 | 3.55 | - | 7.55 |
| YX | 9.59 | 7.27 | - | 22.13 |
| WQ | 11.40 | 9.55 | - | 25.89 |
| PF | 78.90 | 69.28 | 5.54 | 215.59 |
| CH | 121.13 | 164.98 | - | 433.39 |

**Table S5 | Statistics analysis of total flavonols content in petals of six rose cultivars**

|  | Average μg/g FW | Standard deviation | Minimum μg/g FW | Maximum μg/g FW |
| --- | --- | --- | --- | --- |
| YI | 641.90 | 258.68 | 325.92 | 1104.54 |
| GC | 556.56 | 281.07 | 329.43 | 1013.90 |
| YX | 850.54 | 446.89 | 374.08 | 1634.83 |
| WQ | 875.15 | 394.22 | 123.17 | 1444.57 |
| PF | 1203.17 | 597.26 | 573.65 | 2385.92 |
| CH | 443.10 | 109.95 | 323.99 | 599.80 |

**Table S6 | Statistics analysis of total carotenoids content in petals of six rose cultivars**

|  | Average μg/g FW | Standard deviation | Minimum μg/g FW | Maximum μg/g FW |
| --- | --- | --- | --- | --- |
| YI | 18.04 | 16.26 | 2.65 | 47.20 |
| GC | 11.21 | 9.29 | 1.61 | 26.93 |
| YX | 1.20 | 1.40 | 0.27 | 3.80 |
| WQ | 1.71 | 2.16 | 0.00 | 5.95 |
| PF | 0.66 | 0.48 | 0.27 | 1.58 |
| CH | 9.64 | 7.36 | 0.70 | 21.64 |


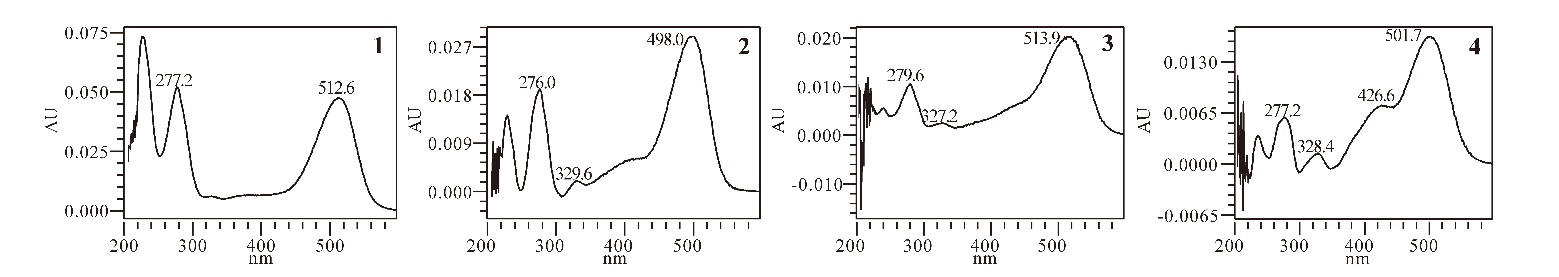


**FIGURE S1 | UV/Vis absorption spectra (λ_max_) of anthocyanins extracted from rose petals.**


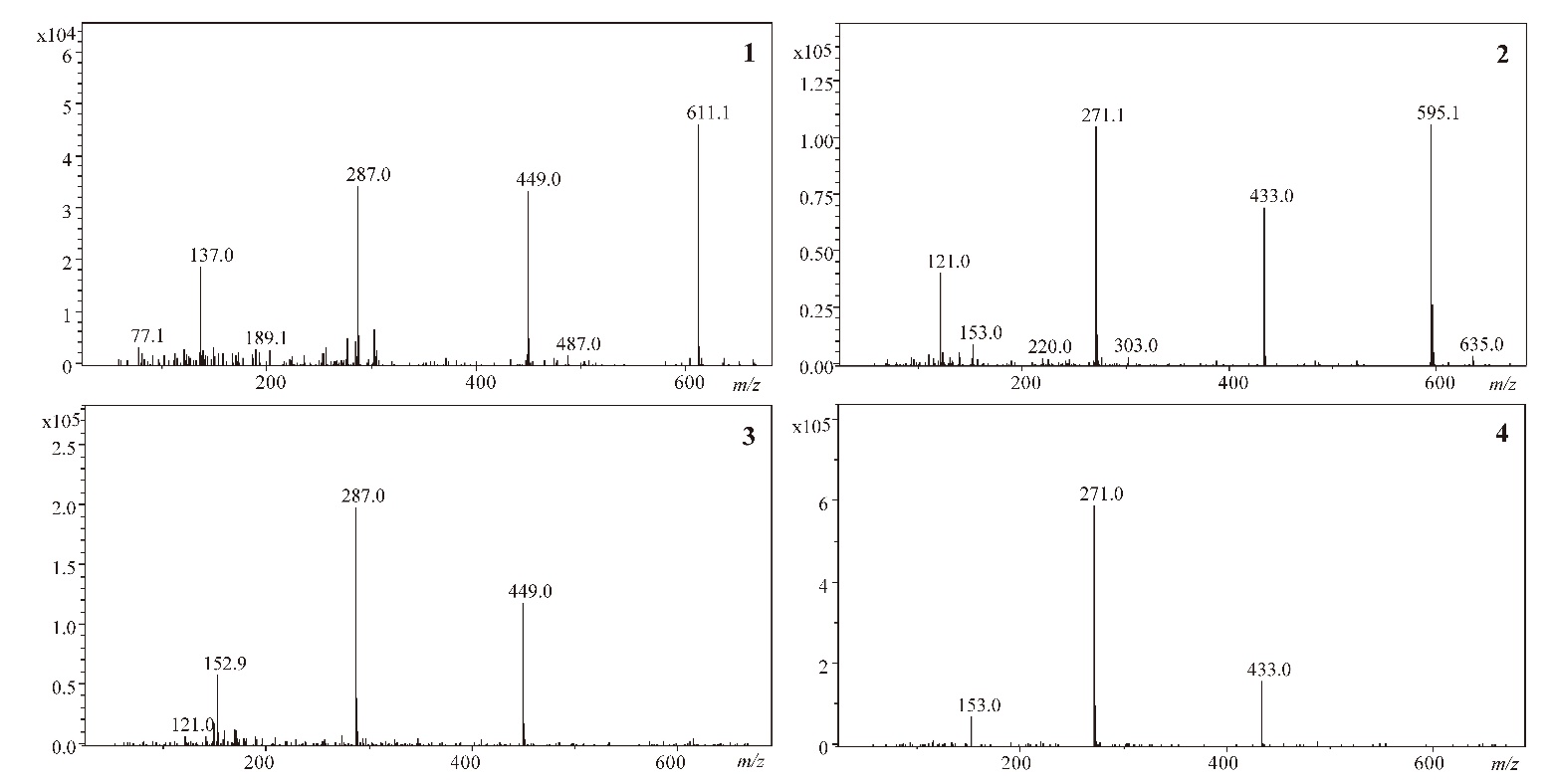


**FIGURE S2 | Mass spectrogram of anthocyanins extracted from rose petals.**
